# XPS and FTIR Studies of Polytetrafluoroethylene Thin Films Obtained by Physical Methods

**DOI:** 10.3390/polym11101629

**Published:** 2019-10-09

**Authors:** Joanna Piwowarczyk, Roman Jędrzejewski, Dariusz Moszyński, Konrad Kwiatkowski, Agata Niemczyk, Jolanta Baranowska

**Affiliations:** 1 West Pomeranian University of Technology, Szczecin, Faculty of Mechanical Engineering and Mechatronics, Institute of Materials Science and Engineering, al. Piastow 19, 70-310 Szczecin, Poland; Agata.Niemczyk@zut.edu.pl (A.N.); Jolanta.Baranowska@zut.edu.pl (J.B.); 2 Łukasiewicz Research Network–PORT Polish Center for Technology Development, ul. Stabłowicka 147, 54-066 Wrocław, Poland; Roman.Jedrzejewski@zut.edu.pl; 3 West Pomeranian University of Technology, Szczecin, Faculty of Chemical Technology and Engineering, Institute of Inorganic Chemical Technology and Environment Engineering, al. Piastow 42, 71-065 Szczecin, Poland; Dariusz.Moszynski@zut.edu.pl; 4 West Pomeranian University of Technology, Szczecin, Department of Mechanics and Fundamentals of Machine Design, Faculty of Mechanical Engineering and Mechatronics, 70-310 Szczecin, Poland; Konrad.Kwiatkowski@zut.edu.pl

**Keywords:** ATR-FTIR, chemical structure, polytetrafluoroethylene thin film, pulsed electron beam deposition, pulsed laser deposition

## Abstract

Two methods—attenuated total reflection Fourier infrared spectroscopy (ATR-FTIR) and X-ray photoelectron spectroscopy (XPS)—have been used to analyze the chemical structure of polytetrafluorethylene (PTFE) thin coatings deposited by pulsed laser (PLD) and pulsed electron beam (PED) ablations. The volume of the analyzed materials is significantly different in these techniques which can be of great importance in the characterization of highly heterogeneous thin films. Optical microscopy, atomic force microscopy (AFM) and scanning electron microscopy (SEM) have been additionally used to examine the coating surface morphology. The studies have shown that in the case of thin polymer coatings deposited by physical methods, the application for chemical structure evaluation of complementary techniques, with different surface sensitivity, together with the use of surface topography imaging, provide unique insight into the film morphology. The results can provide information contributing to an in-depth understanding of the deposition mechanism of polymer coatings.

## 1. Introduction

Polytetrafluoroethylene is a synthetic polymer that is often used as a coating because of its particular combination of chemical and mechanical properties such as flexibility at low temperatures, low coefficient of friction, stability at high temperatures, high chemical resistance to corrosive reagents, insolubility in the majority of organic solvents, long-term weatherability, nonflammability, and hydrophobicity [1,2,3]. Polytetrafluorethylene (PTFE) can be used in many applications in branches such as medicine, bioscience, mechanics, microelectronics, and chemistry [1,4,5]. 

Conventional wet methods for the preparation of thin polymer films, such as spin coating, are often not suitable for PTFE due to its poor solubility in all solvents and non-stick behavior. For these reasons, physical methods such as vacuum evaporation, radio frequency sputtering, plasma evaporation, and ion beam sputtering are of great interest because they are not solvent dependent [6,7,8,9,10]. However, studies indicate that the PTFE coatings obtained using these methods do not preserve the structure of the starting material [6,8,9,10]. 

Pulsed laser deposition (PLD) and pulsed electron deposition (PED) are examples of physical methods that are considered to be promising alternatives to traditional methods of producing very thin films of stoichiometric polymer. PLD and PED methods are dry processes and may be particularly useful in cases where the polymer cannot be processed by conventional thermal or solution techniques [11,12].

For PTFE deposition, the PLD method [1,2,3] has been more widely studied than PED. In most cases, however, use of PLD process results in the deposition of an uneven coating composed of various types of particles. Heitz and Dickinson [13] characterized in detail the particle morphologies of the PTFE coatings that they obtained using the PLD process. They identified four groups of particulates depending on their size and morphology:

- From 10 to 200 nm, probably originating from vapor phase particle growth.
- From 0.5 to 10 µm feathery chains, which are made of aggregated type-I particles.
- Approximately 1 µm ejecta; compact and very symmetric particles, known as droplets.
- Above 4 µm ejecta; fused, open-structure “popcorn-like” material that is the result of the spallation of target “grains” (singly or in clusters), triggered by the shock wave generated by the original thermal pulse accompanying absorption.

Many authors report that the surface quality can be improved by an annealing process, however, this has the disadvantage that the final coating thickness changes after annealing and is therefore poorly predictable, and that partial degradation of the material (yellowing, browning) occurs during annealing. Deposition onto a heated substrate has also been suggested, but the required temperature may be a limitation for some materials [5,14,15]. One reason for the ablation of large particles can be the fact that in most of the processes analyzed, the PTFE targets are obtained by powder pressing with only partial remelting, resulting in PTFE with low molecular weight. The deposition of PTFE coatings by the PED method, in contrast to the PLD method, allows for even and smooth coatings to be deposited [16,17]. The majority of studies, assume that the chemical structure of the deposited film remains unchanged, and this has been confirmed by FTIR or by the preservation of certain typical PTFE characteristics [12,14,18,19].

Chemical structure analysis based on infrared spectroscopy is a very useful technique able to determine the structure of thin films quite accurately as we have shown in our previous work [20]. Nonetheless, when using only one technique for the structure analysis, the limitation of this method has to be taken into account, especially in the case of non-homogeneous coatings. Besides the technical problems arising from the fact that analyzed films are very thin, it is important to be aware that some of the absorption bands may occur at similar or virtually identical wavenumbers, making the interpretation of the spectrum very difficult. This is the case with most of the alkyl halides, also PTFE, because the characteristic absorption bands of their functional groups are in the fingerprint region. In addition, their most dominant and intensive bands (CF2 asymmetric and symmetric stretching bands) have maxima at around 1210 and 1150 cm^−1^, which overlap with the C–C band at about 1240 cm^−1^ [21,22]. A further difficulty in assessing the structure of PTFE based only on IR spectra is that the CF_2_ wagging, bending and rocking vibrations are located in the 640–540 cm^−1^ region undergoing intense changes simultaneously with the crystallinity degree of PTFE [23,24]. 

The unique helical spatial structure of PTFE macromolecules, which results from the presence of fluorine in the main chain, leads to a high thermal resistance, but also to quite high susceptibility to irradiation, resulting in the defluorination of the polymer [25]. However, this phenomenon has not been discussed so far in current research on laser and electron ablation of PTFE. This is probably due to the fact that the absorption bands of defluorinated species of the deposited macromolecules are in the 1300–1150 cm^−1^ wavenumber range, overlapping with CF_2_ absorption bands, which complicates the correct analysis of IR spectra.

Given the above, and our own experience with PLD and PED techniques [16,20,26], the aim of this work was to verify the hypothesis regarding the deposition of stoichiometric PTFE –(*C*F_2_–*C*F_2_)*_n_*– thin films by both PLD and PED techniques, performing detailed chemical structure analysis by infrared spectroscopy in combination with a complementary surface sensitive technique—X-ray photoelectron spectroscopy. Additionally, in view of the “still open discussion” on the mechanism of ablation processes, the films were examined as-deposited (without post-process annealing) in order to determine the effect of PLD and PED on the material. 

## 2. Materials and Methods 

### 2.1. Film Preparation

PTFE coatings were deposited by means of a PED/PLD system (NEOCERA, Inc., Beltsville, MD, USA). The setup consisted of a vacuum chamber, a PEBS-20 pulsed electron source and an excimer laser (Coherent CompexPro 201F; He/Ne; KrF, λ = 248 nm, Santa Clara, CA, USA). 

The PTFE coatings were deposited on monocrystalline Si (100) substrates 10 mm × 10 mm in size. Prior to deposition the substrates were sonically cleaned in an acetone bath, rinsed in acetone and isopropyl alcohol and dried in an air flow. A PTFE bulk disk (Tarflen^®^, P.H.U. SZCZEL-PLAST S.C., Mikołajów, Poland) was used as the target. The chamber was evacuated to 0.1 mPa using nitrogen as the background gas. The PTFE film deposition took place at room temperature and at nitrogen pressures of 0.4, 0.93, and 1.46 Pa for the PED process and 0.13, 0.93, and 1.2 Pa for the PLD process. The deposition time was the same for all PED and PLD processes, corresponding to 5000 pulses at a 5 Hz pulse repetition rate. 

Prior to each deposition the substrate was masked and a pre-ablation conditioning of the target consisting of 2000 pulses was performed in order to clean the target surface and avoid the formation of droplets. The PTFE target was rotated during the deposition process in order to avoid local overheating and transition to the liquid phase. The distance between the target and substrate was set to 80 mm. The electron source (PED) was operating at 12 kV, which corresponded to a pulse energy of 200 mJ and the pulse width was 100 ns. In the case of the laser source (PLD) 20 ns pulses with energy set to 700 mJ were used. 

### 2.2. Film Characterization

The surface topography was examined using optical microscopy (NIKON, Tokyo, Japan), atomic force microscopy (AFM; Veeco NanoScope IVa)—5.0 µm × 5.0 µm images were obtained in contact mode and scanning electron microscopy (SEM; Hitachi SU-70, Tokyo, Japan). The coatings were covered by gold before examination with the SEM. The film thickness was estimated by measuring the step between the coating and an uncoated part of the substrate, which had remained covered during the deposition process. For these measurements a profilometer (Dektak 6M, Veeco) was used with a 1 mg force and 12.5 µm stylus radius. The chemical structures were characterized using Attenuated Total Reflection Fourier Infrared spectroscopy (ATR-FTIR; Lumos, Bruker, Billerica, MA, USA) and X-ray Photoelectron spectroscopy (XPS, PREVAC, Rogów, Poland). For the FTIR analysis, 64 scans at a resolution of 4 cm^−1^ were carried out for each sample. Each spectrum was collected with an air background and corrected for CO_2_ and H_2_O. All spectra presented in the results are after baseline correction and in the wave number range 600–4000 cm^−1^. The X-ray photoelectron spectra were obtained using Al Kα (hν = 1486.6 eV) radiation with a Prevac system equipped with a Scienta SES 2002 electron energy analyzer operating at constant transmission energy (pass energy of 50 eV). Due to the possible PTFE decomposition caused by x-ray radiation the experiment time was limited to 1 h for each sample. The charging effects were corrected by setting a component of C 1s transition corresponding to C–C bonds with a binding energy of 285.0 eV. The quantitative surface composition was calculated assuming an homogeneous distribution of elements in a near-surface layer.

## 3. Results

### 3.1. Surface Morphology and Thickness

**Figure 1:**
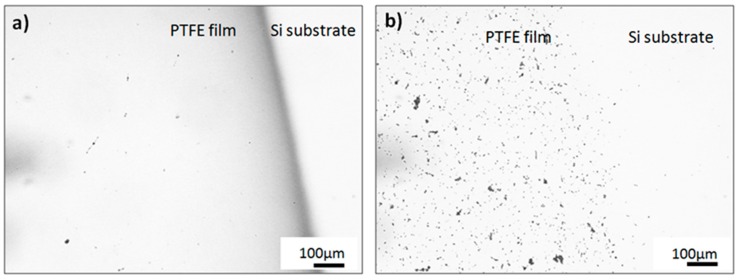
Micrographs of polytetrafluorethylene (PTFE) coatings obtained at 0.93 Pa by (**a**) pulsed electron beam (PED) and (**b**) pulsed laser (PLD) technique; optical microscopy 10×.

Optical microscope images at a magnification of 10 times are presented in Figure 1. It can be seen that the coating obtained by the PED method is very smooth, whilst the surface of the coating obtained by the PLD method is covered by numerous large particulates. The presence and intensity of the latter were independent of the PLD deposition parameters.

**Figure 2:**
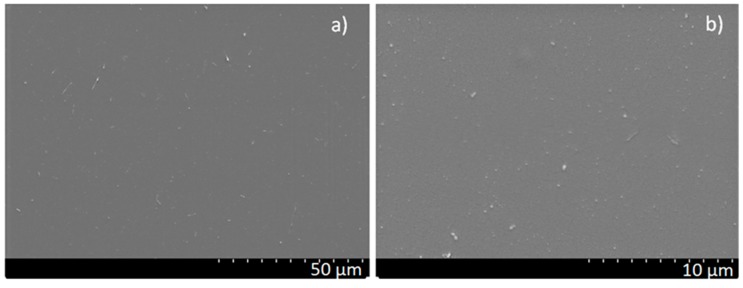
Micrographs of PTFE coatings obtained by PED method at 0.4 Pa at different. magnifications: (**a**) 1000× and (**b**) 5000×; SEM.

**Figure 3:**
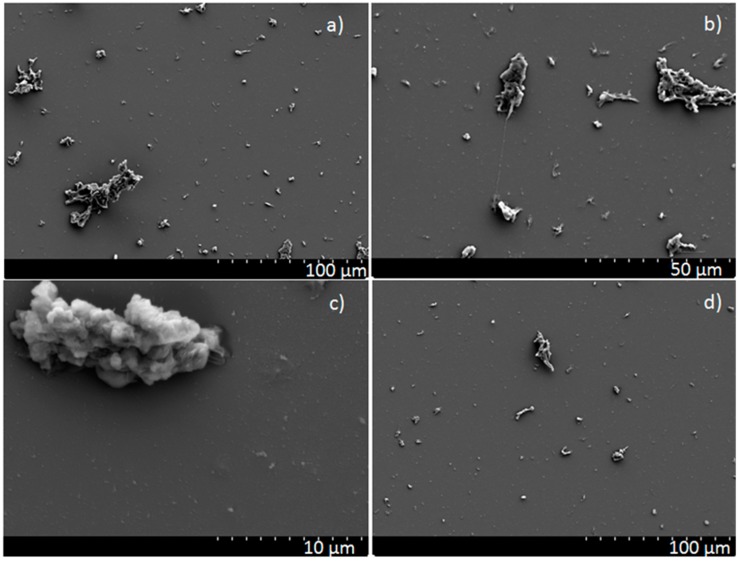
Micrographs of PTFE coatings obtained by PLD method (**a**), (**b**), (**c**) at 0.93 Pa and (**d**) at 0.13 Pa—magnification (**a**) 500×, (**b**) 1000×, (**c**) 5000×, and (**d**) 500×; SEM.

SEM studies confirmed the smooth appearance of the PED coatings (Figure 2); only a few small spherical particulates are present on the surfaces. The morphology of the PLD coatings is much more complex (Figure 3). Numerous particulates are visible, with a patchy and complex structure and with size varying from a few to tens of micrometers. This result, typical for PTFE film deposition by the PLD technique, seems to be independent of the process parameters. This has been confirmed by our own study and also in the literature [15,16,18]. Unfortunately, such a result significantly reduces the quality of the layers, becoming particularly problematic in cases where the substrate cannot be heated to the 340 °C needed to perform post-annealing [1].

**Figure 4:**
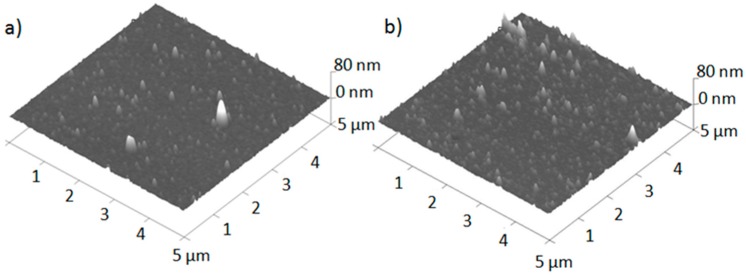
Three dimensional images of the PTFE coatings obtained at 0.93 Pa by (**a**) PED and (**b**) PLD methods; AFM.

In order to examine the film surface between particulates, samples were scanned using atomic force microscopy. The surfaces of the coatings obtained by the PED and PLD methods are shown in Figure 4a,b, respectively. Regardless of the deposition gas pressure and deposition method, the morphology of typical deposits can be described as a continuous film with a grain-like structure and a few small spikes representing particulate matter. Most of the particulates are smaller than 200 nm, with a few exceptions that measured over 500 nm. The number and spatial density of the particulates is higher in the coatings obtained by the PLD method. 

The thickness of the coatings obtained by the PED method was in a 100–200 nm range, depending on the gas pressure used; this was described in detail in our previous work [16] and by other authors [27]. Measurement of the thickness of the coatings obtained by the PLD technique proved very difficult due to the large particulates present on the surfaces, which caused large discrepancies during the measurements. The XPS measurements indicate the presence of a weak silicon signal for PLD formed samples. The mean free path of the electrons corresponding to the Si 2p transition in the PTFE substrate is about 3.5 nm. Therefore, it is supposed that the film thickness for the PTFE layer formed by PLD is low. This shows that the PED method can be considered as much more efficient in the formation of uniform thin coatings (excluding deposited particulates). 

### 3.2. Chemical Structure Characterization

**Figure 5:**
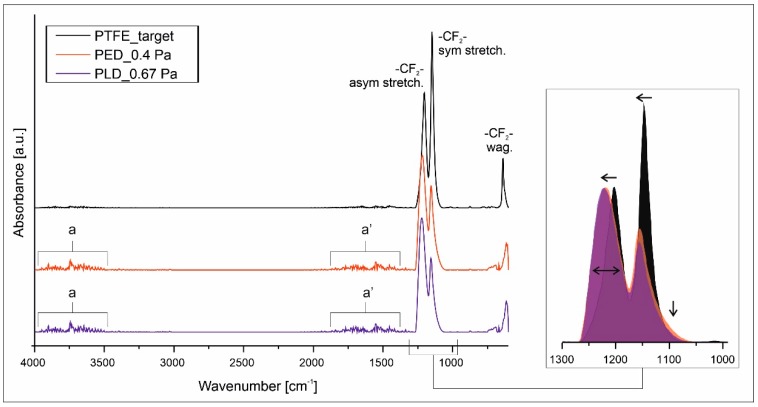
FTIR spectra of the PTFE coatings and target material.

Figure 5 shows the full range and magnification of the 1400–1000 cm^−1^ range FTIR spectrum obtained for the PTFE target material which was used for deposition and examples of typical spectra of the films obtained by the PED and PLD techniques. The PTFE target spectrum typically has two characteristic peaks at 1201 cm^−1^ and 1150 cm^−1^ that are attributed to asymmetrical and symmetrical CF_2_ stretching. A third weaker peak corresponding to the CF_2_ wagging is observed at 642 cm^−1^, which is consistent with literature data [2,11,18,19,28,29].

The FTIR spectra of both types of deposited films indicate no major chemical differences in comparison to the target material, as all main bands are identified close to the maxima of the target. Nevertheless, the magnification of the 1300–1000 cm^−1^ wavenumber range (insert in Figure 5) shows that a more accurate analysis exposes alteration of the chemical structure of the deposited macromolecules.

Considering the main absorption bands of PTFE (i.e. –CF_2_–), it is observed that the ratio of the asymmetric and symmetric stretching bands intensity has been reversed and the absorption maxima have been shifted to higher wavenumbers (1230 and 1155 cm^−1^, respectively). These two changes denote that the primary helix structure of the PTFE macromolecules undergo some transformation as a consequence of the UV and electron radiation [23,30]. Additionally, the observed broadening of the whole absorption area may be attributed to the appearance of some new bands, for example from defluorinated group, that are difficult to recognize without additional data. Two regions with bands of very low intensity—marked by letter a and a′ in Figure 5—remain unidentified. It is difficult to state unambiguously whether they are noise resulting from e.g., a small coating thickness, or whether they indicate the occurrence of absorption bands derived from oxygen groups (COOH and COF groups) or unsaturated groups, formed as a result of free radical reactions or just the moisture. To answer these questions, further structural analysis by the XPS method was carried out.

The elemental composition of the coating surfaces calculated basing on the results of X-ray photoelectron spectroscopy analysis are presented in Table 1. 

**Figure 6:**
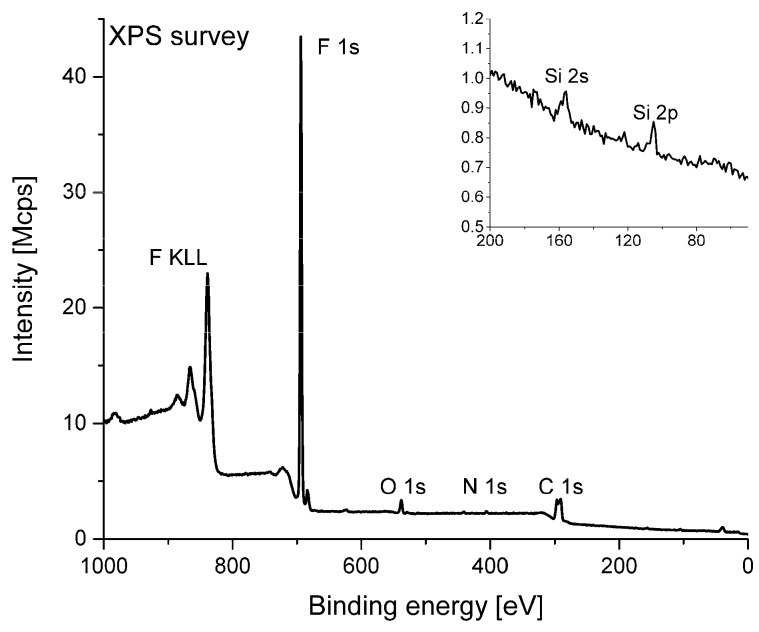
An example XPS survey spectrum acquired for the PTFE sample.

The surface of the samples obtained by the PED method consisted only of fluorine and carbon. The surface of the samples obtained by the PLD method also consisted predominantly of fluorine and carbon, however, some contamination with oxygen and nitrogen atoms was detected for these samples. In two out of the three examined samples prepared by the PLD method an XPS signal of silicon atoms was observed (insert in Figure 6).

The presence of the silicon atoms on the surface observed by XPS may be explained by the low thickness of the PTFE layer deposited on the Si substrate as mentioned in the former chapter. It is possible that during the formation of a very rough PTFE surface some pits in the layer are also formed. As a result, the silicon substrate surface is exposed to the vacuum and detected by XPS analysis. 

The fluorine to carbon ratio calculated as the quotient of the respective surface compositions (in atomic percent) for the target material is about 2.4. This value is higher than the theoretical F/C ratio for PTFE material, considered as a –(*C*F_2_–*C*F_2_)*_n_*– polymer. However, a similar fluorine enrichment of the PTFE surface has been reported previously [25]. The F/C ratio observed for the PED coating is very similar to the one observed for the target material. However, the F/C ratio measured for the PLD coating is less than 2. It is possible that a partial defluorination takes place on the surface of films prepared by PLD. A similar decrease of the F/C ratio has been previously observed for PTFE coatings formed by RF sputtering [31,32].

**Figure 7:**
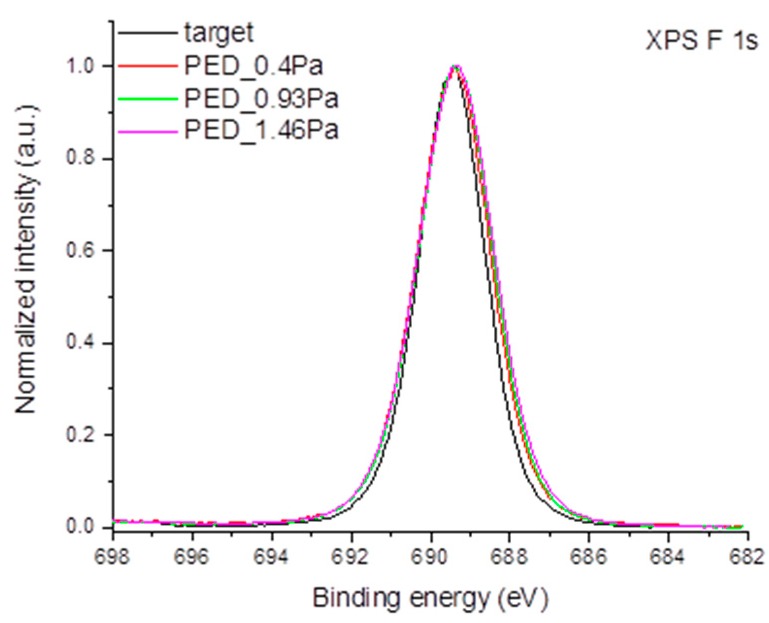
XPS F 1s spectra obtained for the target and PED coated samples.

The chemical composition of the thin films obtained by PED and PLD was analyzed based on high-resolution XPS spectra. The XPS F 1s spectra obtained for the target and the samples prepared by the PED method are presented in Figure 7.

The position of the maximum of the XPS F 1s transition is identical for all samples and is located at a binding energy of 689.4 eV. This binding energy is characteristic for fluorine atoms present in the functional group with covalent C-F bonds such as: –CFR– or –CF_2_– [33]. The XPS F 1s spectra acquired for the sample produced by the PLD method are virtually identical to the ones shown in Figure 7. 

**Figure 8:**
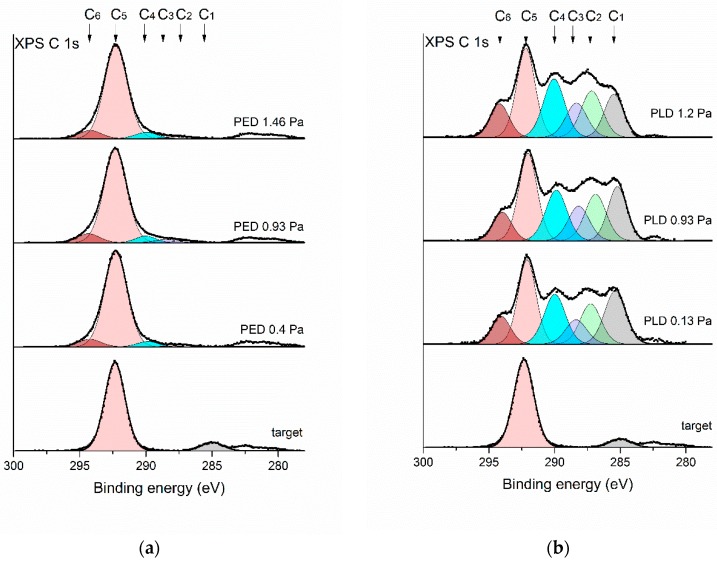
High-resolution XPS C 1s spectra obtained for PTFE target and the samples obtained by (**a**) PED or (**b**) PLD at various pressures.

The XPS C 1s spectrum originating from the target is shown in Figure 8. The maximum of the prominent peak observed for that sample is located at a binding energy of 292.3 eV. This position is characteristic for the –(*C*F_2_–*C*F_2_)*_n_*– bonds which constitute the PTFE structure. A minor peak is observed at a binding energy of 285.0 eV, which is ascribed to adventitious carbon—a contamination of the target surface. These results indicate that the target surface consists of pure PTFE with negligible environmental contamination. Similar XPS results, both for the main peak and a contamination peak, are reported for other analyses of PTFE surfaces [25,34].

In comparison to the target material the envelopes of the acquired XPS C 1s lines vary slightly for the samples prepared by PED and significantly for the samples prepared by PLD. To recognize the chemical transformations of the target material after exposure to the electron beam or laser pulses and its further deposition on the Si substrate, a detailed analysis of the high-resolution XPS spectra was carried out. The envelope of the XPS C 1s spectrum was deconvoluted into several components corresponding to the different chemical environments of carbon atoms. Since the majority of the XPS signal observed during the experiments originated from carbon and fluorine atoms only the presence of different C–F(H) functional groups was considered in the simulation model. Six basic C 1s components was assumed to be sufficient to properly deconvolute the envelopes of all XPS C 1s spectra recorded for the samples formed in both PED and PLD processes. The positions of the C 1s components were based on the binding energy shifts reported in [35]. The model components are characterized in Table 2 and briefly described below. 

Component C1 located at about 285 eV is ascribed to the non-functionalized sp^3^-hybridized carbon atoms observed for hydrocarbons. The component C2 located at about 287 eV represents carbon atoms that are not directly bound with fluorine atoms but which are in the vicinity of other fluorinated carbon atoms in the polymer chain. The component C3, with the maximum of its binding energy at about 288 eV, corresponds to carbon atoms bound to only one fluorine atom or the carbon atoms having no direct bonds with fluorine but located close to the chemical environment with several fluorine atoms. The component C4 is ascribed to carbon atoms located in a polymer chain with several –CFR– groups in a row. The most intense peak observed in all the recorded spectra is located at a binding energy of about 292 eV and is marked as C5. This corresponds to carbon atoms in –(*C*F_2_–*C*F_2_)*_n_*– functional groups in neat PTFE. At the high-energy side of the main maximum a peak at about 294 eV can be discerned. This environment is ascribed to –CF_3_ terminal groups of fluorinated polymer chains and denoted as C6. 

The deconvolution procedure based on the above basic C 1s components was carried out with the application of several additional constraints. The position of the peak maximum was varied by no more than ±0.2 eV. The positions of all C1–C6 peaks given in Table 2 were used for all the considered samples: the target as well as the PED and PLD formed deposits. The full width in half maximum (FWHM) of the model lines was kept between 1.8 and 2.0 eV. 

The positions of the C1–C6 peaks are indicated by arrows in Figure 8. The results of the deconvolution procedure, indicating the fractions of the whole XPS C 1s signal corresponding to the considered chemical environments, are given in Table 3 and are shown as component peaks in Figure 8. 

The deconvolution of the target spectrum indicates that, apart from minor environmental contamination reflected as a small peak at 285 eV, the target material consisted only of –(*C*F_2_–*C*F_2_)*_n_*– functional groups. On the surface of the samples prepared by the PED method (Table 3) the component C1 was not observed at all. This indicates that the adventitious carbon contamination present on the surface of the target is not transferred onto the substrate during the deposition process. The surfaces prepared by the PED process are similar to the pure PTFE. The concentration of the C5 component exceeds 80% of the total XPS C 1s signal. 

For PED, only a small number of CF_3_ groups along with a small amount of defluorinated carbon are recognized, the presence of which can explain the previously described absorption band changes in the 1300–1000 cm^−1^ region of the IR spectra. Nonetheless, the XPS data together with the FTIR results and micrographs of the samples obtained by PED show that the surface chemical composition is virtually identical to the target composition and remains almost pure PTFE. 

The chemical composition of the surface of the coatings produced by the PLD method is very different from that observed for the target and the samples formed by the PED process (compare Figure 8a,b). The envelope of the XPS C 1s transition is complex and extends over a wide range of binding energy from 285 to 294 eV with several prominent local maxima. The deconvolution of these spectra was carried out using an identical model as that described above. The results of this procedure are shown in Table 3. 

The presence of many chemical environments for the carbon atoms indicates that the PLD process results in a substantial defluorination and recombination of C–F functional groups of the PTFE target. Even at the lowest process pressure of 0.13 Pa the signal coming from –(*C*F_2_–*C*F_2_)_*n*_– groups, considered as neat PTFE, is below 30% of the total XPS C 1s signal. The defluorination of the chemical environment of the carbon atoms may be partial as indicated by the presence of C3 and C4 components (–CFR–*C*FR–CFR–or –CH_2_–*C*FR–CH_2_– groups). However, the abundance of C1 and C2 components indicates that in a substantial part of the material deposited during the PLD process, some carbon atoms are totally defluorinated and consequently crosslinked, since the aliphatic hydrocarbon groups are prone to such free radical reactions initiated by physical factors such as UV radiation [36]. These defluorinated atoms constitute approximately 40% of all carbon atoms present in the final product of PLD deposition. Recombination of –(*C*F_2_–*C*F_2_)_*n*_– groups into terminal –*C*F_3_ functional groups is also observed. The internal structure of the XPS spectrum observed for these samples is similar to the spectra observed for sputter-deposited fluorocarbon films [31].

The XPS analysis of the surface of the samples obtained by the PLD process shows that a very prominent degradation of the initial PTFE material occurs. The final composition of these coatings is far from that of expected for a pure PTFE layer. 

The XPS results clearly demonstrate a significant difference in chemical structure between the films obtained by the PED and PLD methods. Moreover, the findings regarding the films obtained by the PLD technique contrast with the results of the FTIR measurements, which did not show very significant changes in the chemical structure for these films. The chemical degradation of the samples formed by PED observed by XPS is negligible and in agreement with the results obtained from FTIR analysis.

XPS is a more surface-sensitive method, therefore some degree of the degradation observed by XPS is presumably located within the upper atomic layers of the coatings. Considering the results obtained for PLD coatings, the difference between the results obtained by XPS and FTIR analysis may be caused by the inhomogeneous surface topography of the PLD coatings, which affects each analysis method in a different way. There was large amount of particulates observed on the surface (Figure 3). During the FTIR measurements the ATR crystal was in close contact with the sample surface, which results in flattening of the protruding particulates. The penetration depth of the IR radiation is of the order of a few micrometers which means that both the thin continuous film and the particulates are within the analyzed volume. Taking into account the size and number of the particulates their volume exceeded the volume of the continuous thin film by at least an order of magnitude. In this way the FTIR spectra are dominated by information originating from the structure of the particulates. In contrast, XPS is contactless and gives information about a very small volume of the material close to the surface, with a thickness of a few nanometers. In the case of morphologically complex surfaces such as those on the PLD coatings, the XPS spectrum contains information originating from the thin continuous film and a thin surface layer on the particulates.

In the light of the results obtained, it can be hypothesized that in the case of laser ablation, large non-degraded particles of material are extracted from the target. However, their transport to the substrate is probably possible due to an expansion of gaseous products of localized degradation, resulting e.g., from the heterogeneity of the PTFE structure [37]. Such a mechanism would be in agreement with the theories regarding the mechanism of polymer transport during laser ablation presented in [38]. Evaporated degraded polymer deposits from the vapor phase in the form of a thin film. This differs from the composition of the starting material and is characterized by a significant loss of fluorine and the presence of oxygen containing compounds, as confirmed by XPS analysis. 

In the case of electron beam deposition, the results obtained would indicate a different transport mechanism. According to the model proposed in [39], ablation can be the result of the evaporation of small pieces of the chain, formed as a result of macromolecule cutting, which then repolymerize on the substrate. While in the case of polymers the term “evaporation” may be controversial, for PTFE this mechanism is particularly plausible due to the presence of two fluorine atoms, thanks to which the formation of linear chains is thermodynamically favored [40,41]. This would explain the very good preservation of the PTFE chemical structure in PED coatings.

A detailed analysis of the ablation mechanisms clearly requires further research, however, it should be emphasized that the proper characteristics of the coatings obtained are crucial for their understanding. 

The issue with different results for different analysis methods does not arise in the subject-related literature. The majority of publications concerning PLD PTFE coatings present FTIR measurements only [12,13,14]. In the work of Smausz at al. [15], the spectra are not included in the XPS results presented and moreover, these results concerned coatings obtained at higher temperature than in the present work, or annealed, which could significantly change the coverage of the surfaces as a result of melting of the particulates. In our experiments owing to the combined use of both ATR-FTIR and XPS it was possible to thoroughly characterize the chemical structure of the PTFE films and the inhomogeneities in the coating morphology. 

## 4. Conclusions

Thin PTFE coatings were successfully deposited by pulsed laser and pulsed electron beam techniques. Significant differences in the surface quality of the coatings were observed depending on the deposition method. PED coatings were smooth with only a few very small spikes, whereas PLD coatings were additionally covered by very large particulates with complex shape. In the case of PED coatings, FTIR and XPS methods confirmed that the chemical structure was preserved. In contrast, PLD coatings were shown to be composed of a thin continuous film made of degraded polymer material and covered with large PTFE particulates spalled off from the target due to localized degradation. The application of two spectroscopic techniques (ATR-FTIR and XPS) provided complementary information about the chemical structure of the PTFE films and allowed the inhomogeneities observed in the coating morphology to be characterized in more detail. They also allowed for a more in-depth analysis of the mechanisms accompanying the ablation process. 

From the experiments it can be concluded that the PED technique has important advantages compared to the PLD method as regards polymer thin film deposition. Most importantly the polymer is stoichiometrically transferred from the target with PED, which is limited in the case of the PLD method. Another important advantage is that the PED method offers the possibility of producing uniform films without the need for post-treatment. 

## Figures and Tables

**Table 1:** Chemical composition of the surface estimated basing on X-ray photoelectron spectroscopy (XPS) survey spectra.

Sample	Carbon	Fluorine	Oxygen	Nitrogen	Silicon	F/C Ratio
Atomic percent						
target	29.7	70.3	-	-	-	2.4
PLD 0.13Pa	33.6	59.2	3.0	0.3	3.9	1.8
PLD 0.93Pa	33.0	63.6	2.3	0.5	0.6	1.9
PLD 1.2Pa	33.1	64.5	1.9	0.5	-	1.9
PED 0.4Pa	29.8	70.2	-	-	-	2.4
PED 0.93Pa	30.3	69.7	-	-	-	2.3
PED 1.46Pa	30.6	69.4	-	-	-	2.3

**Table 2:** Assignment of the C 1s components applied to the fitting procedure of high-resolution XPS C 1s spectra.

Component Number on Fitted C 1s Spectrum	Position of Maximum of Component (eV)	Carbon Group or Structural Unit Corresponding to the Component (Carbon: C)
C1	285.2 ± 0.2	Non-functionalized aliphatic carbons
C2	287.2 ± 0.2	–CH_2_–*C*H_2_–CF_2_–, –CFR–*C*R_2_–CFR–
C3	288.3 ± 0.2	–CH_2_–*C*FR–CH_2_–, –CH_2_–*C*H_2_–CF_3_, *C*–(CFR)_4_
C4	290.0 ± 0.2	–CFR–*C*FR–CFR–
C5	292.1 ± 0.2	–(*C*F_2_–*C*F_2_)*_n_*–
C6	294.2 ± 0.2	–CFR–*C*F_3_–CR_2_–*C*F_3_

**Table 3:** This is a table. Tables should be placed in the main text near to the first time they are cited.

Sample	C1	C2	C3	C4	C5	C6
target	10	-	-	-	90	-
PLD 0.13Pa	20	15	9	19	28	9
PLD 0.93Pa	17	16	12	18	28	9
PLD 1.2Pa	14	16	12	20	27	11
PED 0.4Pa	-	2	-	5	86	7
PED 0.93Pa	-	2	3	5	82	8
PED 1.46Pa	-	2	1	5	84	8
